# Iodine Absorption in Celiac Children: A Longitudinal Pilot Study

**DOI:** 10.3390/nu13030808

**Published:** 2021-03-01

**Authors:** Maurizio Delvecchio, Francesca Bizzoco, Rosa Lapolla, Antonia Gentile, Cinzia Carrozza, Michele Barone, Simonetta Simonetti, Paola Giordano, Vanessa Nadia Dargenio, Fernanda Cristofori, Ruggiero Francavilla

**Affiliations:** 1 Metabolic Disorder and Diabetology Unit, “Giovanni XXIII” Children Hospital, 70126 Bari, Italy; mdelvecchio75@gmail.com; 2 Department of Pediatrics, “Giovanni XXIII” Children Hospital, “Aldo Moro” University of Bari, 70126 Bari, Italy; francescabizzoco.med@gmail.com (F.B.); paola.giordano@uniba.it (P.G.); vane.nadia@gmail.com (V.N.D.); fernandacristofori@gmail.com (F.C.); ruggiero.francavilla@uniba.it (R.F.); 3 Pediatrics Unit, “San Carlo” Hospital, 85100 Potenza, Italy; rosa_lapolla@libero.it; 4 Pediatrics Unit, “Antonio Perrino” Hospital, 72100 Brindisi, Italy; anto.gentile01@gmail.com; 5 UOC Chimica, Biochimica e Biologia Molecolare Clinica, Fondazione Policlinico Universitario “A. Gemelli”, IRCCS, Università Cattolica del Sacro Cuore, 00168 Roma, Italy; cinzia.carrozza@policlinicogemelli.it; 6 Section of Gastroenterology, Department of Emergency and Organ Transplantation, “Aldo Moro” University of Bari, 70125 Bari, Italy; 7 Neonatal Screening Center and Clinical Pathology Unit, “Giovanni XXIII” Children Hospital, 70126 Bari, Italy; ssimonettis@libero.it; 8 Interdisciplinary Department of Medicine-Pediatrics Section, “Aldo Moro” University of Bari, 70121 Bari, Italy

**Keywords:** celiac disease, iodine, thyroid, urinary iodine concentration, endocrine consequences

## Abstract

**Background:** non-autoimmune thyroid disorder is a common finding in celiac patients, more frequent than in the general population. An impairment of iodine absorption has been hypothesized, but it has never been investigated so far. We aimed to evaluate the iodine absorption in children and adolescents with newly diagnosed celiac disease. **Methods:** 36 consecutive celiac patients (age 7.4 years, range 2.4–14.5 years) before starting a gluten-free diet (GFD) were enrolled. We assayed the urinary iodine concentration (UIC) in a 24-h urine sample, at baseline (T0) after 3 (T1) and 12 months (T2) of GFD. **Results:** UIC at T0 was 64 μg/L (IQR 45–93.25 μg/L) with an iodine deficiency rate of 77.8%. UIC was not different according to histological damage, clinical presentation (typical vs atypical); we found no correlation with the thyroid function tests and auxological parameters. UIC was not statistically different at T1 (76 μg/L) and T2 (89 μg/L) vs T0. UIC at T2 was similar between patients with positive and negative anti-transglutaminase antibodies at T2. No patients presented overt hypothyroidism during the study. **Conclusions**: We found that iodine absorption in celiac children is impaired compared to the general population; it increases slightly, but not significantly, during the GFD. We should regularly reinforce the need for a proper iodine intake in celiac disease patients to reduce iodine deficiency risk.

## 1. Introduction

Celiac disease (CD) is an immune-mediated systemic disorder elicited by gluten and related prolamines in genetically susceptible individuals [1]. The critical genetic elements (human leukocyte antigen DQ2 and DQ8), the auto-antigen involved (tissue transglutaminase, tTG), and the environmental trigger (gluten) are well known. The intestinal mucosal damage induced by gluten determines villous atrophy and activation/expansion of B cells responsible for autoantibodies production [2]. 

Autoimmune thyroid disorders (ATD) and non-autoimmune thyroid disorders (NATD) occur in 5–12% of celiac patients [3,4,5,6,7], a figure that is higher than in healthy controls [7,8,9]. In particular, NATD has been reported to be 3-times more frequent in CD than in general population [6,7]. It has been hypothesized that NATD in CD is secondary to a decreased thyroid hormones synthesis, induced either by an iodine organification defect or by a functional hypothalamic-pituitary disturbance consequent to isolated malnutrition [10,11]. Indeed, gluten withdrawal is often followed by the normalization of thyroid function [5,7].

Iodine is an essential micronutrient for the synthesis of thyroid hormones, and the first step is the absorption in the small intestine [12]. The observation that the mucosal recovery secondary to the gluten-free diet (GFD) is followed by normalization of thyroid function has reinforced the idea that iodine malabsorption contributes to NATD in CD [6]; however, this hypothesis has never been investigated so far. 

In the present study, we aimed to evaluate iodine absorption in children and adolescents newly diagnosed with celiac disease before starting the GFD and up to one year later.

## 2. Materials and Methods 

### 2.1. Study Design 

We performed a longitudinal study between February 2017 and May 2019 at the Pediatric Department of the University Hospital of Bari (Italy), a tertiary referral centre for the diagnosis and follow-up of endocrinological and gastroenterological disorders in our region. We recruited children and adolescent with a new diagnosis of CD. The celiac patients were followed-up for 12 months, and symptoms, auxological data, thyroid function tests, urinary iodine concentration (UIC), and anti-tTG were recorded at baseline (T0), after 3 (T1), and 12 months (T2) of GFD. 

### 2.2. Subjects

We recruited 36 children diagnosed with CD based on serologic tests and a duodenal biopsy according to the European Society for Pediatric Gastroenterology, Hepatology, and Nutrition (ESPGHAN) criteria [13]. The inclusion criteria were: 1. Diagnosis of CD according to ESPGHAN guidelines; 2. Willingness to join the study; 3. Age at recruitment <18 years. The exclusion criteria were 1. GFD before the diagnosis of CD; 2. Previous diagnosis of thyroid disease; 3. ATD at recruitment or during the 12-month follow-up; 4. Any disease that could affect thyroid function (i.e., chronic liver or renal disease, autoimmunity or malignancy); 5. Medication that could influence serum thyroid-stimulating hormone (TSH), free thyroxine (fT4), and free triiodothyronine (fT3).

The study adhered to the Declaration of Helsinki and the protocol was approved by the local ethical committee in Bari (Study number: 5200; protocol number: 26989CE); all patients/guardians/controls gave their informed consent prior to inclusion in the study.

### 2.3. Methods 

Data collection included clinical history, growth assessment and thyroid function tests. Height (H) and weight (W) in underwear were measured and the nutritional status evaluated by the body mass index (BMI). 

Serum TSH, fT4, fT3 and antibodies against thyroperoxidase (anti-TPO) and thyroglobulin (anti-TG) were assayed. Biochemical assays were performed using commercial kits (Dimension EXL integrated chemistry system LOCI Module Siemens, Erlangen, Germany) with immunoenzymatic (TSH, fT4, and fT3; TSH normal range 0.3/3.6 μg/L, fT4 normal range 0.70/1.80 ng/dL, fT3 normal range 2.2/4.2 pg/mL) and immunoradiometric techniques (anti-TPO and anti-TG antibodies). Hypothyroidism was defined as TSH above the normal values, and classified as subclinical if TSH ≤10 μg/L (levothyroxine replacement not required) or overt if TSH >10 μg/L; levothyroxine replacement was started if TSH was confirmed >10 μg/L after 4 weeks. 

Serum IgA concentrations, tTG-IgA, and endomysial antibodies (EMA) were tested. Quantitative detection of tTG was assessed by an ELISA test (ORGENTEC Diagnostika; Mainz, Deutschland; cut-off value: >10 AU) and EMA-IgA by indirect immunofluorescence, using monkey’s oesophagus sections as substrate (Euroimmun Italia Diagnostica Medica SRL; Padova, Italia; cut-off >1:10). IgA levels were assayed by nephelometry in all subjects. No patients showed selective IgA deficiency (defined as serum IgA <0.05 g/L). Human leukocyte antigen (HLA) class II typing (DQA1*02:01, DQA1*03, DQA1*05, DQB1*02, DQB1*03:01/03:04, DQB1*03:02/03:05, DRB1*03, DRB1*04, DRB1*07, DRB1*11, DRB1*12) was performed by PCR sequence-specific oligonucleotide using DQ-CD Typing Plus (DiaGene, Palermo, Italy) [14].

Patients with positive serological tests for CD underwent upper endoscopy with multiple duodenal biopsies according to ESPGHAN criteria to confirm the diagnosis [13]. The same pathologist graded all biopsies specimens [15]. After recruitment, all celiac patients started GFD. 

Iodine absorption was assessed by the UIC in 24 h-urine samples. The urinary iodine excretion is a very sensitive indicator of iodine intake since iodine is absorbed by the small intestine and mostly excreted by the kidney. All subjects recruited in the study were instructed by an experienced dietitian to guarantee a well-balanced diet with an adequate amount of iodized salt (daily intake of 3–5 g of salt containing 30 ppm of iodine) in keeping with the World Health Organization (WHO) program [16] for at least ten days before the measurement. Dietary recall about iodine intake and GFD was performed at each visit. Patients and controls received a proper container and were invited to collect urine from the second nicturition of the day to the first of the following morning. When the collection was returned, the urine was shaken, and two samples were obtained and immediately stored and frozen at −20 °C for later analysis. The assay was performed in the Chemistry and Clinical Biochemistry Laboratory, Catholic University School of Medicine, Rome, Italy. Urine iodine levels were analyzed by colorimetry (LTA s.r.l., Milan, Italy) using a spectrophotometric procedure based on the Sandell-Kolthoff reaction, in which iodate ion acts as a catalyst in the reduction of ceric ammonium sulphate (yellow color) to the cereus form (colorless) in the presence of arsenious acid. The specimens were treated using ammonium persulfate in advance to eliminate interfering contaminants. 

In patients >6 years of age at recruitment, the iodine intake was classified as: insufficient if UIC <100 μg/L; adequate between 100 and 199 µg/L; above requirements between 200 and 299 μg/L; and excessive when ≥300 μg/L [16].

On the basis of WHO guidelines, the patients were classified on the basis of age at recruitment as 0–5 years (suggested minimal iodine intake 90 μg/day), 6–12 years (recommended iodine minimal intake 120 μg/day), and >12 years (suggested minimal iodine intake 150 μg/day) [16]. 

### 2.4. Statistical Analysis

Height and BMI were expressed as standard deviation score (SDS) [17]. The statistical analysis was performed with IBM SPSS Statistics v20.0 computer software for Mac. Data were reported as median and interquartile range (IQR) and analyzed by non-parametric tests. The differences between frequencies were evaluated by the Chi-Square (χ^2^) test and the differences among groups by the Mann-Whitney U test or the Kruskal-Wallis H test as appropriate. The difference between paired groups was evaluated by the Wilcoxon test. Correlations were evaluated by Spearman’s correlation coefficient. Finally, patients were categorized into tertiles for UIC and BMI to compare the other continuous variables. A *p*-value < 0.05 (2-sided level) was considered statistically significant.

## 3. Results

### 3.1. Patients Features

We recruited ten males (27.8%) and 26 females (72.2%) with a median age of 7.4 years (IQR 4.6/9.8 years), height −0.45 SDS (−1.21/0.98 SDS), and BMI −0.25 SDS (−0.96/0.32 SDS). All the enrolled patients showed mucosal atrophy [15 patients with grade B1 (villous to crypt ratio less than 3:1 with still detectable villi), and 21 with grade B2 (villi no longer detectable) according to Corazza-Villanacci classification] [15]. Twenty-one patients (58%) presented with typical symptoms (diarrhea, and/or bloating and/or weight loss) while fifteen with atypical symptoms or were asymptomatic. Two patients (5.5%) had short stature (H < −2 SDS) and 2 patients (5.5%) were underweight (BMI SDS < −2 SDS). TSH was 2.31 μg/L (1.85/3.06 μg/L), fT4 1.03 ng/dL (0.99/1.13 ng/dL), and fT3 4.18 pg/mL (3.61/4.22 pg/mL). Seven patients (19.4%) presented subclinical hypothyroidism at recruitment, and none of them overt hypothyroidism (highest TSH 8.47 μg/L, fT4 normal in all patients) (Table 1).

Fifteen patients were 0–5 years old, seventeen 6–12 years old, and four older than 12 years old.

### 3.2. Findings at T0

The UIC was 69 μg/L (45/93.25 μg/L) in the study group and 72 μg/L (45/120 μg/L), 68 μg/L (47/89.5 μg/L) and 80 μg/L (41.75/130.25 μg/L) in patients 0–5, 6–12 and older than 12 years, respectively (*p* = ns among the 3 age groups). Seventeen patients out of 21 who were older than 6 years at recruitment (80.9%) were iodine insufficient. No significant correlations were found between UIC and TSH, fT4, fT3, age at recruitment, H SDS, and BMI SDS in the study group. 

**Figure 1:**
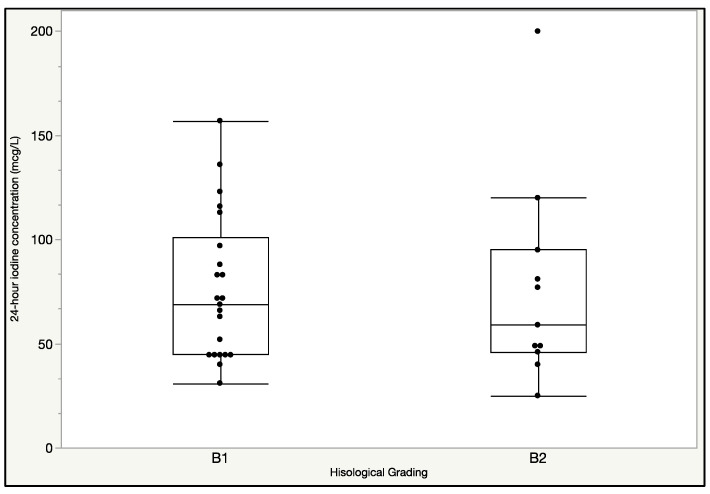
24-h urinary iodine concentration (UIC) boxplot and scatter plot in patients with B1 [72 (45/88) μg/L] and B2 [63 (44.5–105) μg/L]) grading at T0 (*p* = ns).

Celiac patients with grade B1 had similar UIC, 72 μg/L (45/88 μg/L), to patients with grade B2, 63 μg/L (44.5/105 μg/L) (Figure 1; *p* = ns). TSH, fT4, fT3, H SDS, and BMI SDS were not different between patients with grade B1 and B2, but the formers were younger [5.2 (4.4/7.5) years] than the latter [9.3 (6.25/11.1) years] (*p* = 0.028).

The UIC at diagnosis was not different between patients with typical symptoms and patients with atypical symptoms [72 μg/L (44.5/101.0 μg/L) vs 66.5 μg/L (45.2/86.5 μg/L); *p* = ns].

No statistical difference was found between the 28 patients with iodine deficiency and the 8 patients with adequate iodine intake as regards TSH [2.27 μg/L (1.70/3.00 μg/L) vs. 2.62 μg/L (2.04/4.00 μg/L), respectively], fT4 [1.02 ng/dL (0.99/1.11 ng/dL) vs. 1.07 ng/dL (0.99/1.46 ng/dL), respectively], and fT3 [4.18 pg/mL (3.69/4.35 pg/mL) vs. 3.78 pg/mL (3.25/4.25 pg/mL), respectively].

H, BMI SDS, and thyroid hormone levels were not statistically different among the three tertiles for UIC. Similarly, UIC, H, and thyroid hormone levels were not statistically different among the three tertiles for BMI SDS. 

### 3.3. Findings at T1 

Data were available for 28 patients (two patients did not attend the visit, three patients asked to drop out from the study, three patients did not complete the urine collection). The UIC was 76 (60.25/105) μg/L, not different from the value at T0 of 69 (45.25/93.25) μg/L. 

The UIC was 97 μg/L (74/167 μg/L) and 63 μg/L (39/82 μg/L) in patients 0–5 and 6–12 years old, respectively (only 2 patients older than 12 years). Fourteen patients out of 17 older than 6 years of age at recruitment (82.3%) were iodine insufficient. No significant correlations were found between UIC and TSH, fT4, fT3, age at recruitment, H SDS, and BMI SDS in the study group.

TSH was 2.19 μg/L (1.70/3.46 μg/L), fT4 1.05 ng/dL (0.97/1.14 ng/dL), and fT3 4.23 pg/mL (3.60/4.66 pg/mL). Six patients (20.7%) presented subclinical hypothyroidism and one hypothyroidism (TSH 10.6 μg/L) without treatment as TSH decreased spontaneously after one month. Thyroid function tests were not statistically different from the values at T0. The TSH and fT3 levels were not different between the 20 patients with iodine deficiency and the 8 patients with adequate iodine intake, while fT4 was lower in patients with iodine deficiency (*p* = 0.033). 

UIC at T1 did not correlate with UIC at T0 and was not different between celiac patients with grade B1 and B2 at diagnosis. 

### 3.4. Findings at T2

Data were available for 23 patients (1 patient did not attend the visit, one patient asked to drop out from the study, three patients did not complete the urine collection). The UIC was 89 μg/L (48/124 μg/L), not statistically different from the value at T0 of 59 μg/L (44/95 μg/L) and at T1 of 81 μg/L (62/118 μg/L). 

UIC was 112 μg/L (75/136 μg/L) and 60 μg/L (37/123 μg/L) in patients 0–5 and 6–12 years old, respectively (only 2 patients older than 12 years). UIC was not statistically different as compared to T0 and T1 in each age subgroups. Ten patients out of 14 older than 6 years of age at recruitment (71.4%) were iodine insufficient. 

TSH was 2.03 μg/L (1.52/3.50 μg/L), fT4 1.00 ng/dL (0.95/1.19 ng/dL), and fT3 4.08 pg/mL (3.72/4.38 pg/mL). Four patients (16%) presented subclinical hypothyroidism and none overt hypothyroidism. Thyroid function tests were not different from the values at T0 and at T1. The TSH, fT4 and fT3 levels were not different between the 16 patients with iodine deficiency and the 7 patients with adequate iodine intake. 

UIC at T2 was correlated with UIC at T0 (r2 = 0.427, *p* = 0.042) and at T1 (r2 = 0.516, *p* = 0.017) and was not different between celiac patients with grade B1 and grade B2 at diagnosis. 

**Figure 2:**
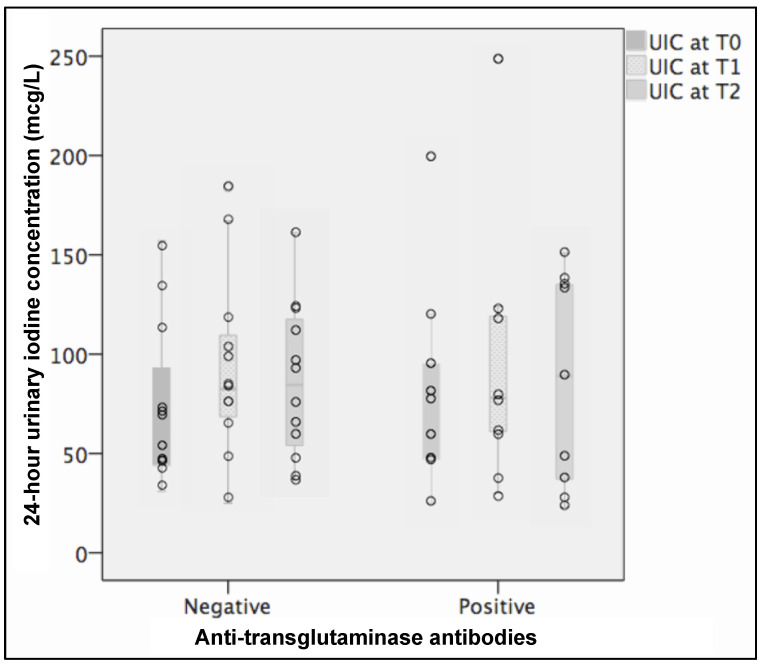
24-h urinary iodine concentration (UIC) boxplot and scatter plot in patients with positive (10 patients) and negative (13 patients) celiac serological tests at T2. *p*-value was not significant in both groups. In patients with negative serological celiac tests UIC was 60 μg/L (44/103.5 μg/L) at T0, 82.5 μg/L (65.5/113.3 μg/L) at T1, and 84.5 μg/L (51/120.3 μg/L) at T2. In patients with positive serological celiac tests UIC was 77 μg/L (46.5/107.5 μg/L) at T0, 78 μg/L (50/121.5 μg/L) at T1, and 89 μg/L (32/136.5 μg/L) at T2.

Anti-tTG tests were still positive in 10 patients (43.5%) and UIC was not different between patients with positive and negative tests. In patients with positive celiac tests, UIC was 68 μg/L (44.5/101.2 μg/L) at T0 and 102 μg/L (34.5/135.7 μg/L) at T2 (*p* = ns), while in patients with negative celiac tests it was 52 μg/L (44.0/93.0 μg/L) at T0 and 76 μg/L (54.0/117.5 μg/L) at T2 (*p* = ns). Figure 2 displays the UIC at T0, T1, and T2.

Dietary recall showed an appropriate iodine intake [16] and strict compliance to the GFD [18] at each time point.

Age, UIC, TSH, fT4 and BMI were not different between patients who dropped out and who did not drop out of the study (Table S1). 

## 4. Discussion

The present study, first in the CD literature, shows that children and adolescents newly diagnosed with CD present iodine deficiency and that this state improve, even if not significantly after one year of GFD. The iodine deficiency appears much more evident in school age (about 80% at recruitment, after one year of GFD) as compared to pre-school age children. At diagnosis, the median UIC in our patients appears strikingly lower than the median value of 125 μg/L found in Italian schoolchildren in the same period, even if patients 11–13 years old [19]. Iodine absorption slightly increases during the first year of dietary treatment, even if the increase does not reach statistical significance. 

In Italy, since 2005, a law (n 55/2005) introduced a nationwide program of iodine prophylaxis through the use of iodized salt (30 ppm of a gram of salt). Ever since the General Direction of Food Safety and Nutrition at the Italian Ministry of Health and the experts of the Italian National Observatory for Monitoring Iodine Prophylaxis (OSNAMI) intensified the national informative campaigns with the slogan “less salt but iodized”; their efforts successfully led to iodine sufficiency [19]. 

Iodine plays a central role in the physiology of the thyroid gland, where it exerts its role through two iodine containing-hormones, T3 and T4. The dietary requirement of iodine is determined by T4 production without stressing the thyroid iodide trapping mechanism or TSH levels. We ingest iodine in several chemical forms, mostly reduced to iodide (I−) in the gut [20]. Dietary iodide is actively taken up in the small intestine through the Na+/I− symporter (NIS) [12], a glycoprotein located in the basolateral membrane of the thyroid follicular cells and at the apical surface enterocytes of the small intestine [21], which actively accumulates iodine. Iodine enters the circulation as plasma inorganic iodide, which is cleared from circulation by the thyroid, to synthesize the thyroid hormones, and by the kidney, to eliminate the excess. All the steps in thyroid hormones biosynthesis are stimulated by TSH and inhibited by excess iodine [22].

The kidney excretes about 90% of the absorbed iodine with urine within 48 h after intake, and thus the urinary excretion is very reliable to evaluate the iodine intake. In this view, the iodine deficiency was more evident at recruitment in patients 6-12-year-old (median UIC 68 μg/L, suggested minimal iodine intake 120 μg/L) and >12-year-old (median UIC 80 μg/L, recommended minimal iodine intake 150 μg/L). In these patients, UIC was similar after one year of GFD. In patients 0–5 years the median UIC was 72 μg/L at recruitment and 112 μg/L after one year of GFD (suggesting a minimal iodine intake of 90 μg/L), indicating that in this age group the iodine intake is more appropriate than in older patients and that the GFD is beneficial in improving the iodine absorption, even if the increase is not statistically significant as compared to baseline. 

Among the methods to monitor the iodine intake [23], the 24-h UIC is the most widely used measurement to give a precise estimation of the iodine intake [24,25]. At the same time, UIC from spot samples is recommended for population assessment and monitoring of iodine interventions globally [16,23]. In this view, we decided to evaluate the iodine absorption by measuring the UIC in 24-h urine samples. To reduce biases as much as possible, an experienced dietician gave proper dietary recommendations to guarantee a well-balanced diet with an adequate amount of iodized salt. At baseline, we performed the urine collection 10 days after using iodized salt (30 ppm of iodine), which is the primary intervention strategy for iodine deficiency control and prevention, to reduce the risk of deficiency in iodine intake and to standardize the iodine intake. It might be argued that the window of dietary intake was too short for replenishing depleted body stores at recruitment, although data from literature [19] show that basically, our school children are iodine sufficient. 

Thyroid disease is a frequent finding in CD [9], at least 3-fold higher than in healthy controls [7,26,27]. The most frequent etiology is autoimmune (3–6); however, the incidence of NATD is higher in CD patients than in controls [7]. In our cohort, the prevalence of NATD was around 15–20%, and none of the patients developed overt hypothyroidism. It has been hypothesized that a decreased synthesis of thyroid hormones, due to iodine organification defect or to functional hypothalamic-pituitary impairment secondary to malnutrition, may account for that [7]. We have previously ruled out that pituitary autoimmunity could cause changes in TSH levels [28]. Cassio et al. in a longitudinal study in 135 CD children with at least 3-years of follow-up, found that 13% of patients presented NATD at CD diagnosis, confirming previous data and suggesting the existence of a difference as a yet unknown mechanism [6]. Since the small intestine is the site for iodine absorption, they hypothesized that gliadin induced mucosal enteropathy could impair iodine absorption, thus contributing to the etiology of NATD. Iodine absorption occurs throughout the length of the small intestine. Although we do not have data on the extent of the enteropathy, it is known from two different studies on celiac adults, using video capsule endoscopy (VCE), that around 60% of CD patients at diagnosis have extensive enteropathy from the duodenum into the jejunum. In contrast, only 30% had villus changes confined to the duodenum [29,30]. Both studies showed conflicting results on the correlation of the extent of the enteropathy with symptoms severity: Murray et al. [29] didn’t find any association, while Rodonotti et al. [30] showed that patients with entire small bowel enteropathy presented severer (although not significant) symptoms than those with changes limited to the proximal part. Follow-up VCE showed that a GFD for more than 6 months was able to restore intestinal mucosa starting from the distal part [29].

The hypothesis that impaired iodine absorption might be responsible for altered thyroid hormones metabolism has never been proved. Our data rule out that iodine deficiency in CD at diagnosis is associated with higher TSH levels. Still, our study group can be considered too small to draw a final conclusion. It is likely that different factors, such as iodine deficiency and inflammatory cytokines, may interplay affecting the pituitary-thyroid axis. 

We did not find any difference in UIC according to the clinical presentation (typical vs atypical CD). This may be explained by the presence of villous atrophy in all the patients. UIC was not different between patients with partial villous atrophy (B1) as compared to patients with total villous atrophy (B2) (Figure 1), and this might be secondary to the patchy pattern of duodenal enteropathy. We found a progressive, although not significant, increase of UIC after 3 and 12 months of GFD as compared to baseline irrespective of serological tests for CD at one year (Figure 2). It is possible to speculate that a complete anatomical/functional recovery of intestinal mucosa requires a longer period of GFD. This point should be considered for optimal nutritional counselling aiming at an appropriate intake of iodized salt. Celiac patients frequently develop thyroid disorders, and it is well acknowledged that a correct iodine intake in the general population may prevent these disorders. This evidence supports the practical advice to support iodine intake recommendation during nutritional counselling. 

We also tested the hypothesis if the nutritional status could affect the pituitary-thyroid axis, but we did not find any difference regarding TSH across the BMI tertiles. We believe that the mucosal damage does not affect thyroid hormones metabolism by reducing iodine absorption considering that the mechanisms of iodine absorption are redundant. Therefore, the impact of gastrointestinal disorders, such as CD, on iodine homeostasis has to be considered negligible, as suggested by the absence of iodine deficiency in patients with short bowel syndrome or previous malabsorptive surgical procedures [31]. 

Recent data suggest that focusing on selenium metabolism in celiac patients may provide innovative insight. Selenium is absorbed by the duodenum and highly present in the thyroid cells as selenoproteins. A possible reduction of selenium absorption, never evaluated in CD patients, could exert a role in the pathogenesis of NATD. The link between gut microbiota and selenium metabolism is quite strong, as selenium may be actively taken up by the intestinal microbes, causing a reduction of its bioavailability even in the presence of redundant mechanisms of absorption [31]. Imbalances in the intestinal microbiota of patients with CD, mainly characterized by increased *Bacteroides* spp. and decrease of *Bifidobacterium* spp. have been shown [32,33]. 

This paper has some limitations, which deserve comments. First, the relatively small sample size might not have the power to identify iodine deficit correctly; therefore, our findings warrant confirmatory studies in larger cohorts. Second, we recruited only children and adolescents, and thus it could be debated that our results may not be relevant in adulthood.

On the other hand, we think that our paper has two crucial strengths. First, to the best of our knowledge, this is the first study assaying iodine excretion in celiac patients after proper dietary recommendations. Second, the UIC was assayed on a 24-h sample that, at present, is the gold standard to evaluate iodine absorption although, the collection of daily urinary output is a burden for patients.

## 5. Conclusions

In conclusion, our data suggest that school age celiac patients present at diagnosis iodine deficiency that partially recovers after one year of a gluten-free diet. This stresses the need for proper dietetic advice on a possible long-term iodine supplementation, especially in these age group patients. Further studies in larger cohorts and hopefully over a more extended study period could be of great help to confirm or deny our findings to better understand the mechanism underlying iodine absorption in celiac patients. 

## Figures and Tables

**Table 1:** Features of the patients at diagnosis of celiac disease (T0), after 3 (T1) and 12 (T2) months of gluten-free diet

	Study Group		
	T0 (36 Patients)	T1 (28 Patients)	T2 (23 Patients)
**Age (years)**	7.4 (4.6/9.8)	7.6 (5/9.8)	8.5 (5.6/11.6)
**Gender**	10 M, 26 F	8 M, 20 F	6 M, 17 F
**UIC (μg/L)**	64 (45/93.25)	76 (60.25/105)	89 (48/124)
**TSH (μg/L)**	2.31 (1.85/3.06)	2.19 (1.70/3.46)	2.03 (1.52/3.50)
**fT4 (ng/dL)**	1.03 (0.99/1.13)	1.05 (0.97/1.14)	1.00 (0.95/1.19)
**fT3 (pg/mL)**	4.18 (3.61/4.22)	4.23 (3.60/4.66)	4.08 (3.72/4.38)
**tTG-IgA** **(titer range, AU)**	Positive in all patients (31.9/>200)	Positive in 14 patients (50%) (10.1/>200)	Positive in 10 patients (43.5%) (12.1/>200)
**EMA-IgA**	Positive in all patients	n.a.	n.a.

Data are reported as median and interquartile range. n.a.: not available; UIC: urinary iodine concentration; tTG-IgA: anti-transglutaminase IgA; EMA-IgA: anti-endomysial IgA. Data are displayed as median (interquartile range).

## Data Availability

The data presented in this study are available on request from the corresponding author. The data are not publicly available due to privacy policy.

## Supplementary Materials

The following are available online at https://www.mdpi.com/2072-6643/13/3/808/s1, Table S1. Characteristics of patients who completed the study vs patients who dropped out. No statistical differences were found between the 2 groups. Data are displayed as median (interquartile range).

## References

[1] Fasano A., Catassi C. (2005). Coeliac disease in children. Best Pract. Res. Clin. Gastroenterol..

[2] Green P.H., Cellier C. (2007). Medical progress: Celiac disease. N. Engl. J. Med..

[3] Kowalska E., Wasowska-Krolikowska K., Toporowska-Kowalska E. (2000). Estimation of antithyroid antibodies occurrence in children with coeliac disease. Med. Sci. Monit..

[4] Oderda G., Rapa A., Zavallone A., Strigini L., Bona G. (2002). Thyroid autoimmunity in childhood celiac disease. J. Pediatr. Gastroenterol. Nutr..

[5] Ansaldi N., Palmas T., Corrias A., Barbato M., D’Altiglia M.R., Campanozzi A., Baldassarre M., Rea F., Pluvio R., Bonamico M. (2003). Autoimmune thyroid disease and celiac disease in children. J. Pediatr. Gastroenterol. Nutr..

[6] Cassio A., Ricci G., Baronio F., Miniaci A., Bal M., Bigucci B., Conti V., Cicognani A. (2010). Long-term Clinical Significance of Thyroid Autoimmunity in Children with Celiac Disease. J. Pediatr..

[7] Sategna-Guidetti C., Volta U., Ciacci C., Usai P., Carlino A., Franceschi L., Camera A., Pelli A., Brossa C. (2001). Prevalence of thyroid disorders in untreated adult celiac disease patients and effect of gluten withdrawal: An Italian multicenter study. Am. J. Gastroenterol..

[8] Elfström P., Montgomery S.M., Kämpe O., Ekbom A., Ludvigsson J.F. (2008). Risk of thyroid disease in individuals with celiac disease. J. Clin. Endocrinol. Metab..

[9] Sun X., Lu L., Yang R., Li Y., Shan L., Wang Y. (2016). Increased incidence of thyroid disease in patients with celiac disease: A systematic review and meta-analysis. PLoS ONE.

[10] Carter J.N., Corcoran J.M., Eastman C.J., Lazarus L. (1974). Effect of severe, chronic illness on thyroid function. Lancet.

[11] Farthing M.J., Rees L., Edward C.R., Byfield P.G., Himsworth R.L., Dawson A.M. (1982). Thyroid hormones and the regulation of thyroid function in men with coeliac disease. Clin. Endocrinol..

[12] Nicola J.P., Basquin C., Portulano C., Reyna-Neyra A., Paroder M., Carrasco N. (2009). The Na+/I− symporter mediates active iodide uptake in the intestine. Am. J. Physiol. Cell Physiol..

[13] Husby S., Koletzko S., Korponay-Szabó I.R., Mearin M.L., Phillips A., Shamir R., Troncone R., Giersiepen K., Branski D., Catassi C. (2012). European society for pediatric gastroenterology, hepatology, and nutrition guidelines for the diagnosis of coeliac disease. J. Pediatr. Gastroenterol. Nutr..

[14] Buyse I., Decorte R., Baens M., Cuppens H., Semana G., Emonds M., Marynen P., Cassiman J. (1993). Rapid DNA typing of class II HLA antigens using the polymerase chain reaction and reverse dot blot hybridization. Tissue Antigens.

[15] Corazza G.R., Villanacci V. (2005). Coeliac disease. J. Clin. Pathol..

[16] World Health Organization (2007). Assessment of the Iodine Deficiency Disorders and Monitoring Their Elimination: A Guide for Programme Managers.

[17] Cacciari E., Milani S., Balsamo A., Spada E., Bona G., Cavallo L., Cerutti F., Gargantini L., Greggio N., Tonini G. (2006). Italian cross-sectional growth charts for height, weight and BMI (2 to 20 yr). J. Endocrinol. Investig..

[18] Biagi F., Bianchi P.I., Marchese A., Trotta L., Vattiato C., Balduzzi D., Brusco G., Andrealli A., Cisaro F., Astegiano M. (2012). A score that verifies adherence to a gluten-free diet: A cross-sectional, multicentre validation in real clinical life. Br. J. Nutr..

[19] Olivieri A., Trimarchi F., Vitti P. (2020). Global iodine nutrition 2020: Italy is an iodine sufficient country. J. Endocrinol. Investig..

[20] Zimmermann M.B. (2009). Iodine deficiency. Endocr. Rev..

[21] Ravera S., Reyna-Neyra A., Ferrandino G., Amzel L.M., Carrasco N. (2017). The Sodium/Iodide Symporter (NIS): Molecular Physiology and Preclinical and Clinical Applications. Annu. Rev. Physiol..

[22] Cavalieri R.R. (1997). Iodine metabolism and thyroid physiology: Current concepts. Thyroid.

[23] Zimmermann M.B., Andersson M. (2012). Assessment of iodine nutrition in populations: Past, present, and future. Nutr. Rev..

[24] Vejbjerg P., Knudsen N., Perrild H., Laurberg P., Andersen S., Rasmussen L.B., Ovesen L., Jørgensen T. (2009). Estimation of iodine intake from various urinary iodine measurements in population studies. Thyroid.

[25] Montenegro-Bethancourt G., Johner S.A., Stehle P., Neubert A., Remer T. (2015). Iodine status assessment in children: Spot urine iodine concentration reasonably reflects true twenty-four-hour iodine excretion only when scaled to creatinine. Thyroid.

[26] Meloni A., Mandas C., Jores R.D., Congia M. (2009). Prevalence of Autoimmune Thyroiditis in Children with Celiac Disease and Effect of Gluten Withdrawal. J. Pediatr..

[27] Norström F., Van Der Pals M., Myléus A., Hammarroth S., Högberg L., Isaksson A., Ivarsson A., Carlsson A. (2018). Impact of Thyroid Autoimmunity on Thyroid Function in 12-year-old Children with Celiac Disease. J. Pediatr. Gastroenterol. Nutr..

[28] Delvecchio M., De Bellis A., Francavilla R., Rutigliano V., Predieri B., Indrio F., De Venuto D., Sinisi A.A., Bizzarro A., Bellastella A. (2010). Anti-pituitary antibodies in children with newly diagnosed celiac disease: A novel finding contributing to linear-growth impairment. Am. J. Gastroenterol..

[29] Murray J.A., Rubio-tapia A., Van Dyke C.T., Brogan D.L., Knipschield M.A., Lahr B., Rumalla A., Zinsmeister A.R., Gostout C.J. (2008). Mucosal Atrophy in Celiac Disease: Extent of Involvement, Correlation with Clinical Presentation, and Response to Treatment. Clin. Gastroenterol. Hepatol..

[30] Rondonotti E., Spada C., Cave D., Pennazio M., Riccioni M.E., De Vitis I., Schneider D., Sprujevnik T., Villa F., Langelier J. (2007). Video capsule enteroscopy in the diagnosis of celiac disease: A multicenter study. Am. J. Gastroenterol..

[31] Virili C., Centanni M. (2017). “With a little help from my friends”—The role of microbiota in thyroid hormone metabolism and enterohepatic recycling. Mol. Cell. Endocrinol..

[32] Di Cagno R., Rizzello C.G., Gagliardi F., Ricciuti P., Ndagijimana M., Francavilla R., Guerzoni M.E., Crecchio C., Gobbetti M., De Angelis M. (2009). Different fecal microbiotas and volatile organic compounds in treated and untreated children with celiac disease. Appl. Environ. Microbiol..

[33] Cenit M.C., Olivares M., Codoñer-Franch P., Sanz Y. (2015). Intestinal microbiota and celiac disease: Cause, consequence or co-evolution?. Nutrients.

