# Supplementary material for: Iodine Absorption in Celiac Children: A Longitudinal Pilot Study
Source: Nutrients. 2021 Mar 1;13(3):808. doi: 10.3390/nu13030808 (PMC7998751; doi:10.3390/nu13030808)
Supplement: Supplementary file 1 [file nutrients-13-00808-s001.pdf]

Supplementary table S1. Characteristics of patients who completed the study vs patients who dropped out.

	**Drop out group**	**Not drop out group**
Age at recruitment (years)	7.5 (4.6/10.6)	7.2 (4.6/9.6)
UIC (mcg/l)	59 (44/95)	82 (56/105)
TSH (mcg/l)	2.27 (1.07/4.07)	2.54 (2.02/2.98)
fT4 (ng/dl)	1.03 (0.99/1.09)	1.05 (0.96/1.53)
fT3 (pg/ml)	4.19 (3.62/4.38)	4.05 (3.56/4.27)
BMI (SDS)	-0.1 (-1.1/0.4)	-0.4 (-0.9/0.0)
